# How to write a neurology case report

**DOI:** 10.1186/s13256-016-0867-x

**Published:** 2016-04-06

**Authors:** Richard A. Rison

**Affiliations:** University of Southern California Keck School of Medicine, Los Angeles County Medical Center, Medical Director PIH Health Hospital-Whittier Stroke Program, Medical Director PIH Health Hospital-Whittier Non-Invasive Vascular Laboratory, Neurology Consultants Medical Group, 12401 Washington Blvd., Whittier, CA 90602 USA

## Abstract

Neurology case reports have a long history of transmitting important medical information across many generations for the improvement of patient care. Case reports contribute much to the physician’s knowledge base from which treatment hypotheses and ideas form. Elements of a modern case report, as presented in the CARE (CAse REport) guidelines, include the abstract, introduction, case presentation, discussion, conclusion, patient’s perspective, and consent statement. The sections are described here, as well as the application of CARE guidelines to a published neuromuscular case report. Writing case reports offer an ideal opportunity for neurologists to publish interesting case findings and carry on the tradition of neurologic case reporting.

## Introduction

**Fig. 1:**
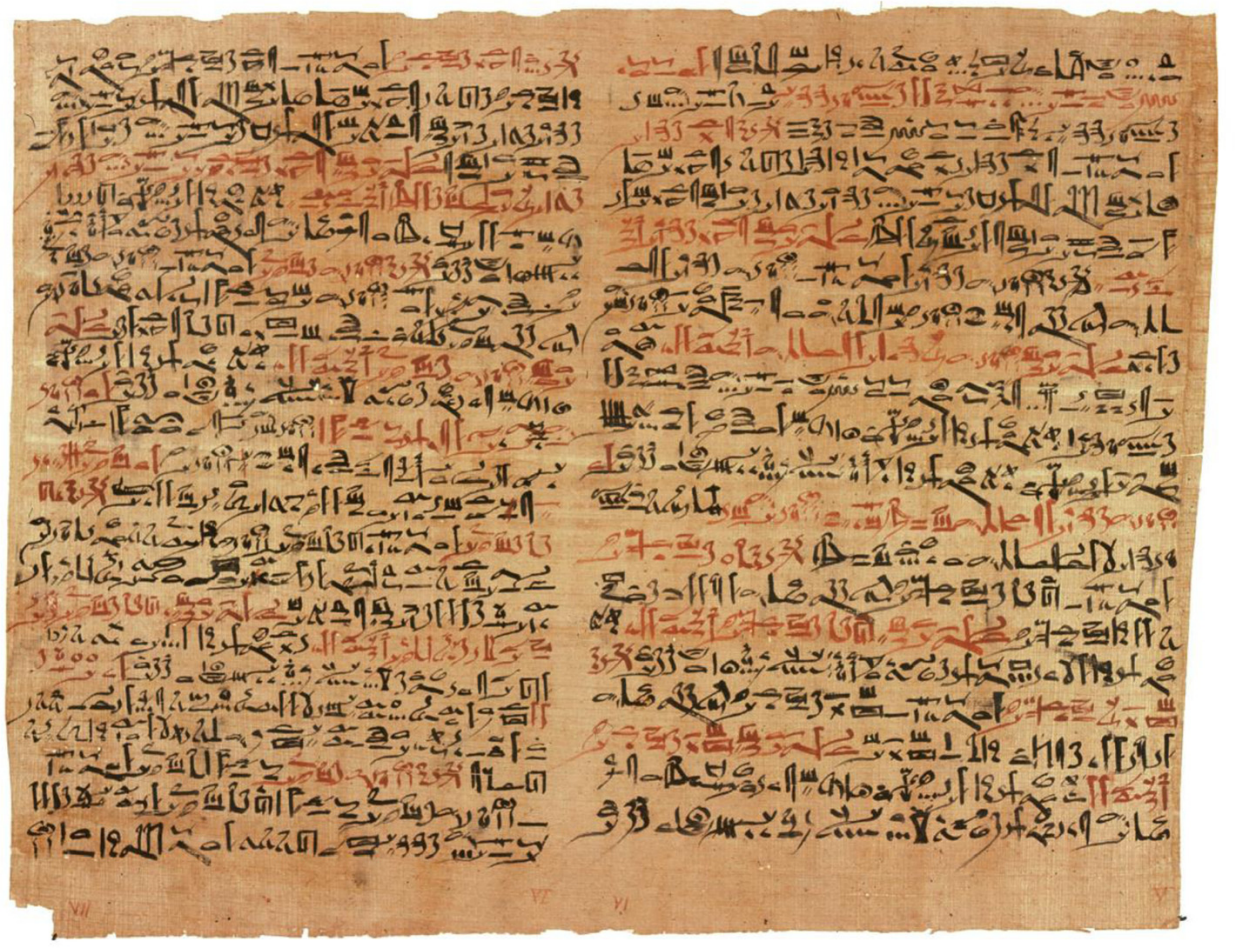
Edwin Smith surgical papyrus, Case 20, c2800 BC

The rich history of neurology case reporting in the medical sciences traces back many generations to the time of Hippocrates (c. 460 B.C.) and, even earlier, to papyrus records of the ancient Egyptians (c. 1600 B.C.) [1, 2]. An early description of aphasia was written by an Egyptian surgeon actually over 4000 years ago (See Fig. 1, Edwin Smith surgical papyrus, Case 20, c2800 BC) [2]. Neurology case reports throughout the centuries have contributed to our understanding of many disease entities. Hippocrates encouraged observation and was himself a keen observer. He was among the first to describe cerebrovascular disease, noting “when persons in good health are suddenly seized with pains in the head and straightaway are laid down speechless and breathe with stertor, they die in seven days when fever comes on” [3]. This author, who sees many patients with acute cerebrovascular disease, finds his characterization of subarachnoid hemorrhage apt to this day.

The time-honored tradition of case reporting forms a cornerstone of medicine. Case reports reflect experiences of busy clinicians and provide the foundation upon which medical knowledge is built. Information accumulated from large studies and controlled trials contributes much to advancements in treatment. Nevertheless, during the course of day-to-day doctor-patient interactions, imagination and intuition often come into play to help arrive at a diagnosis or treatment plan; it is here that case reports, alongside interactions with experienced colleagues and personal experience, contribute greatly to the knowledge base from which ideas and hypotheses form [4].

The case reporting tradition began simply as personal communications between colleagues about unique and interesting patients encountered in their respective practices. The value of the reports has been consistently recognized, and the communication has evolved from anecdotal notes to a standardized format that allows indexing, categorization, and rapid dissemination of knowledge to a broad medical audience [5]. Modern case reports typically include a title, abstract, introduction, case presentation, discussion, conclusion, and references.

Even as the format evolves, the most important element remains the patient and his or her story, and constructing the case report begins with the patient. The process starts with the patient assessment, followed by discussions with colleagues, clinical investigations, treatment if possible, and follow-up examinations. Over the course of time, roughly 6 months at least but sometimes longer depending on the neurologic condition, along with multiple visits, the clinical course develops and a case report may take shape [6].

When an interesting or challenging condition arises in the hospital or private office setting, discussions with colleagues, both within neurology and across other specialties, is often the first step in the investigation. Particularly intriguing cases, or those that require an urgent transfer, may prompt reaching out to local university colleagues [6]. Beyond informal discussions, further research may lead to medical literature and case database searches. Helpful internet literature reference sites include UpToDate [7] and PubMed [8]. The availability of case reports helps tremendously in researching unique cases. BioMed Central (BMC) continues to acknowledge the value of case reports to the scientific literature through publishing the BMC series, a group of open access, peer-reviewed journals spanning most areas of biological and clinical research and where the clinical research journals provide a platform for case reports alongside research articles, and the *Journal of Medical Case Reports* (JMCR), the first international medical journal devoted to publishing case reports from all clinical disciplines [9, 10].

Medical list servers and online communities may be beneficial as well, such as Neurolist and The American Academy of Neurology (AAN) for discussion forums specific to neurology, and SERMO®, which encompasses most specialties [11–13]. To seek input from colleagues through more traditional, pre-Internet routes, visiting teaching grand rounds at a local university, poster presentations, and discussions with professors giving lectures at local seminars are helpful.

Once it has been determined, through discussions and research, that the case at hand presents a potential contribution to medical knowledge, assembling the pieces of the manuscript begins.

## Abstract

The abstract provides enough information for readers to determine whether to continue to read the entire report. It should be a condensed version of the full report with the same main sections. The background explains the importance of the case and whether this is the first report of its kind. The case presentation includes the most important elements of the case, including the patient’s age, sex, and ethnic background. The conclusion states the learning points and clinical impact for a particular specialty or for a broader medical audience [14].

## Introduction

Much of the work that led to the decision to write a case report can be distilled in the introduction to explain the background of the case, including the disorder, usual presentation and progression, and an explanation of the presentation if it is a new disease. If it is a case discussing an adverse drug interaction, details of the drug’s common use and previously reported side effects should be detailed in the introduction. A brief literature review should give an overview of the case from the nonspecialist view. The introduction ends by stating briefly the central message of the report. A concise, salient introduction attracts a reader’s attention and serves as a sales pitch to entice the reader to continue [6].

## Case presentation

All relevant details (except information that identifies the patient) go into the case presentation. The description should be chronological beginning with patient characteristics (for example, age, gender, ethnicity, and occupation), the presenting concerns, and past interventions. Salient history often includes significant family, occupational, and other social history, any significant medication taken or allergies, and any comorbidities. A description of the physical examination starts with the vital signs presented at the examination followed by a detailed neurological examination and pertinent investigations and results.

This section also describes the diagnostic focus and assessment, including laboratory testing, imaging results, questionnaires, referral diagnostic information, diagnostic challenges and reasoning, as well as any prognostic indications. The therapeutic focus and assessment is detailed in the types of interventions, such as pharmacologic, surgical, preventive, lifestyle, or self care. A description of the clinical course encompasses follow-up and outcomes assessments, including intervention modifications, adherence to the intervention, adverse effects, and patient- as well as clinician-reported outcomes [15].

For case series, each patient has a separate description. The presentation strikes a balance between conciseness and offering enough details for the reader to establish his or her own conclusions.

## Discussion

Information that adds context to the case or explains specific treatment decisions often comprise the discussion section, which is optional for JMCR. This section compares and contrasts the case report to published literature, including a brief summary of recent literature and contemporary references. The rationale for the conclusions is given along with ways the case may be generalized to a wider population.

## Conclusions

A succinct explanation of the main conclusions of the case report and their importance and relevance, whether to a particular specialty or to a broader clinical audience, are described here. An explanation of how the information advances our knowledge of a disease etiology or drug mechanism, for example, is given. A concise conclusion with clear take-home messages is most effective.

## Patient’s perspective

As medicine becomes more person-centered, the voice of the individual patient gains more importance in contributing to clinical decisions and medical education. Unique to JMCR, this optional section for the patient’s perspective adds an important new dimension to the traditional case report.

## Consent

Most journals, including all within the BMC portfolio, require a consent section, which states the patient has given his or her informed consent for the case report to be published. If the individual described in the report is a minor, or unable to provide consent, the consent must be given by parents or legal guardians. If the patient is deceased, then consent must be obtained from next-of-kin. The consent statement in the manuscript should reflect this.

**Fig. 2:**
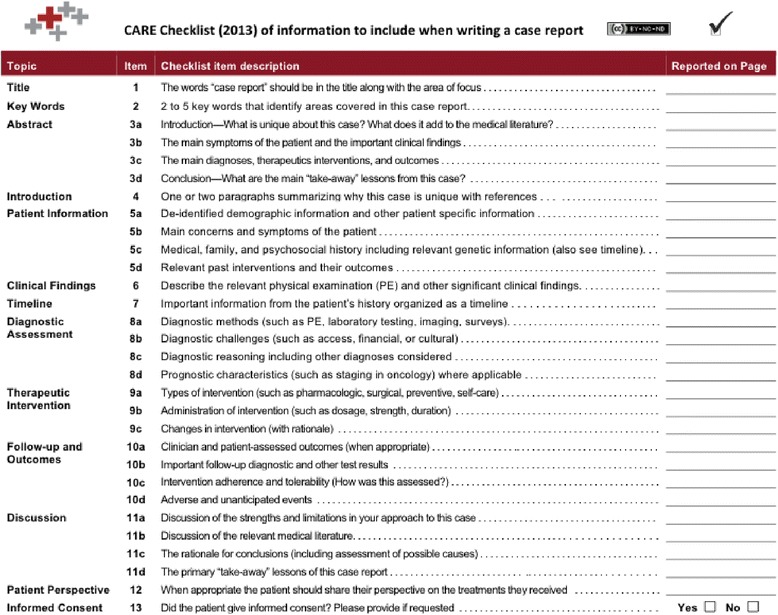
CARE (CAse REport) checklist

Case reports typically contain the sections described above, but there has been wide variability on specific criteria for publication, and, as such, quality may be variable. The CARE (CAse REport) guidelines address the important issue of standardizing case report formats. The primary items of the CARE checklist, shown in Fig. 2, are title, key words, abstract, introduction, patient information, clinical findings, timeline, diagnostic assessment, therapeutic interventions, follow-up and outcomes, discussion, patient perspective, and informed consent [15]. The CARE website describes in detail these elements and provides a summary checklist. The CARE guidelines aim to provide an international, general, non-journal-specific framework for completeness and transparency for published case reports, balancing adequate detail with concise writing [14].

## Example of a neurological case report

**Fig. 3:**
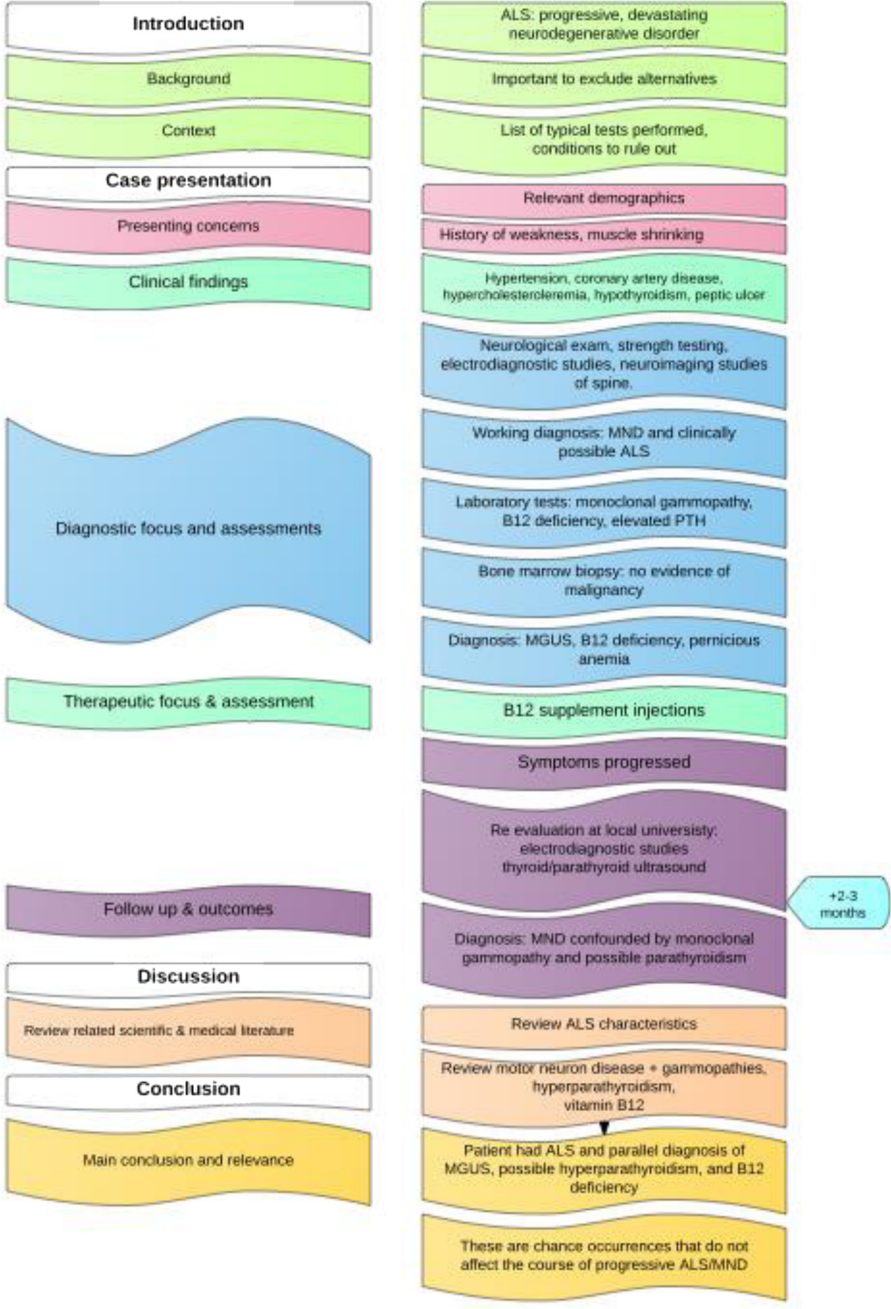
CARE (CAse REport) checklist components mapped to elements in an example neurologic case report

Figure 3 provides an illustration of the CARE guidelines represented in a case report. The report, “Amyotrophic lateral sclerosis-motor neuron disease, monoclonal gammopathy, hyperparathyroidism, and B12 deficiency: case report and review of the literature” [16] describes a Caucasian man who presented with a history of progressive weakness. The diagnosis of motor neuron disease was confounded by monoclonal gammopathy, possible hyperparathyroidism, and B12 deficiency. Due to the gravity of an amyotrophic lateral sclerosis (ALS) diagnosis, all efforts to exclude alternative diagnoses were made. In this case, the occurrence of the confounding factors did not affect the course of progressive ALS. The flow chart diagrams the specific elements provided in the CARE checklist for writing a case report, and outlines the presentation of the patient to a neurology private practice office and subsequent referral to a local university neuromuscular colleague for a second opinion and confirmation of diagnosis.

Now widely accepted within published medical literature, case reports offer a valuable source of information for the medical profession and provide an opportunity for disseminating important clinical observations. By adhering to the CARE guidelines, a solid case report can be assembled. As another checklist, this “rule of C’s” may be helpful: Is it *C*lear*, C*oncise, and *C*oherent? Did you obtain *C*onsent? Was the care of your patient *C*ompetent and *C*ompassionate [6]? The growth of electronic medical journals has expanded the opportunities for publishing case reports. This is an ideal venue for neurology residents, fellows, and practicing attendings to publish and carry on the tradition of neurologic case reporting, adding to the medical literature to help present and future patients.
